# Methods to Generate Innovative Research Ideas and Improve Patient and Public Involvement in Modern Epidemiological Research: Review, Patient Viewpoint, and Guidelines for Implementation of a Digital Cohort Study

**DOI:** 10.2196/25743

**Published:** 2021-12-23

**Authors:** Gloria A Aguayo, Catherine Goetzinger, Renza Scibilia, Aurélie Fischer, Till Seuring, Viet-Thi Tran, Philippe Ravaud, Tamás Bereczky, Laetitia Huiart, Guy Fagherazzi

**Affiliations:** 1 Deep Digital Phenotyping Research Unit, Department of Population Health Luxembourg Institute of Health Strassen Luxembourg; 2 Diabetes Australia Melbourne Australia; 3 Diabetogenic Melbourne Australia; 4 Luxembourg Institute of Socio-Economic Research Esch/Alzette Luxembourg; 5 Centre of Research in Epidemiology and Statistic Sorbonne Paris Cité National Institute of Health and Medical Research (INSERM), French National Institute for Agricultural Research (INRA) Université de Paris Paris France; 6 Centre d’Epidémiologie Clinique, Hôpital Hôtel-Dieu, Assistance Publique-Hôpitaux de Paris Paris France; 7 European Patients’ Academy on Therapeutic Innovation Brussels Belgium

**Keywords:** patient and public involvement, workshops, surveys, focus groups, co-design, digital cohort study, digital epidemiology, social media, mobile phone

## Abstract

**Background:**

Patient and public involvement (PPI) in research aims to increase the quality and relevance of research by incorporating the perspective of those ultimately affected by the research. Despite these potential benefits, PPI is rarely included in epidemiology protocols.

**Objective:**

The aim of this study is to provide an overview of methods used for PPI and offer practical recommendations for its efficient implementation in epidemiological research.

**Methods:**

We conducted a review on PPI methods. We mirrored it with a patient advocate’s viewpoint about PPI. We then identified key steps to optimize PPI in epidemiological research based on our review and the viewpoint of the patient advocate, taking into account the identification of barriers to, and facilitators of, PPI. From these, we provided practical recommendations to launch a patient-centered cohort study. We used the implementation of a new digital cohort study as an exemplary use case.

**Results:**

We analyzed data from 97 studies, of which 58 (60%) were performed in the United Kingdom. The most common methods were workshops (47/97, 48%); surveys (33/97, 34%); meetings, events, or conferences (28/97, 29%); focus groups (25/97, 26%); interviews (23/97, 24%); consensus techniques (8/97, 8%); James Lind Alliance consensus technique (7/97, 7%); social media analysis (6/97, 6%); and experience-based co-design (3/97, 3%). The viewpoint of a patient advocate showed a strong interest in participating in research. The most usual PPI modalities were research ideas (60/97, 62%), co-design (42/97, 43%), defining priorities (31/97, 32%), and participation in data analysis (25/97, 26%). We identified 9 general recommendations and 32 key PPI-related steps that can serve as guidelines to increase the relevance of epidemiological studies.

**Conclusions:**

PPI is a project within a project that contributes to improving knowledge and increasing the relevance of research. PPI methods are mainly used for idea generation. On the basis of our review and case study, we recommend that PPI be included at an early stage and throughout the research cycle and that methods be combined for generation of new ideas. For e-cohorts, the use of digital tools is essential to scale up PPI. We encourage investigators to rely on our practical recommendations to extend PPI in future epidemiological studies.

## Introduction

### Background

Patient and public involvement (PPI) in research is defined as “research being carried out ‘with’ or ‘by’ members of the public rather than ‘to,’ ‘about,’ or ‘for’ them” [1]. PPI means that patients or the public are actively involved in the research process rather than being included only as participants. Public engagement is the 2-way process of engagement activities that benefits both the researcher and the public [2].

Research that involves patients and the public can reduce the mismatch between what matters to patients and what is actually being done in the research [3]. A waste of research resources can be generated when the needs of those likely to use the research results, such as patients and caregivers, are not taken into account [4]. PPI can contribute to the identification and selection of high-priority research questions, planning and performing of more focused research, and improvement of participants’ enrollment in clinical trials [5]. Ultimately, this can result in a higher societal benefit through better use of resources for research. PPI improves the quality of the study and makes research more relevant [6].

Involve, a UK-funded program, aims to improve the quality of research through the integration of PPI throughout the research cycle (identification and prioritization, commissioning, design and management, implementation, dissemination, implementation, and evaluation). Involve has published a report with guidelines to help researchers start new projects when they intend to include PPI in their projects [7].

Digital epidemiology has the same objectives as epidemiology, which are the observation of disease patterns, their evolution, and the causes of these patterns to improve population health and prevent diseases, but digital epidemiology uses digital data [8]. A digital or e-cohort study can integrate data that were not generated for the research (social media and registries) or were generated with digital tools (wearables, sensors, smartphone technologies, and e-questionnaires through web platforms) [9]. Before starting any epidemiological, clinical, or population-based study, researchers need to choose the best methodology to incorporate PPI throughout the project [10]. However, although there are approaches to integrate PPI in a research project, we think that there are no clear recommendations of which methods are the most appropriate, in particular with respect to the launch of cohort studies with digital sources of data.

Although PPI is recommended in research projects, this involvement is often not described or is incompletely reported [11]. A reason for underreporting may be to avoid describing an unsuccessful PPI attempt or that there was no involvement [12]. In addition, there is some evidence that PPI is seldom used in many countries [13]. In the case of cohort studies and, in particular, e-cohorts, we believe that this insufficient involvement may be due to a lack of knowledge of the methods, barriers, or facilitators to apply PPI. In addition, we think that there is a need to have concrete and clear examples for applying PPI in this type of study.

### Objectives

The aims of this study are to (1) review methodologies used to include PPI in research, (2) provide the viewpoint of a final user of research results, and (3) provide practical guidelines and recommendations about how to initiate and run an e-cohort study with PPI based on the review and the point of view of a patient advocate.

## Methods

This work entails 3 parts: a narrative review about methodology and description of PPI, a viewpoint of a patient advocate, and a case study with practical guidelines and recommendations, illustrated by the implementation of a digital cohort study.

### Review

Data for this review were identified by searches of PubMed, Google Scholar, targeted websites about PPI, reports, and existing PPI guidelines, as well as Google Search and references from relevant studies. We used the following search terms: “patient and public participation,” “patient engagement,” “patient involvement,” “consumer involvement,” “community involvement,” “participatory health research,” “community based research,” “research ideas,” “co-writing,” “coproduction,” “co-design,” “cohort study,” “e-cohort,” and “longitudinal study.” We included original studies describing PPI methods using the Involve definition with at least one type of PPI in the study. We included information on studies published in English from 2000 to the present.

### Viewpoint of a Patient Advocate

We used relevant definitions from the European Patients’ Academy on Therapeutic Innovation for patients. We defined patients as individuals with personal experience of the disease, caregivers as individuals supporting a patient, and patient advocates as individuals representing large numbers of patients with a specific disease [14].

We invited a patient advocate to present her perspective and expectations regarding PPI in the context of diabetes research and the use of digital tools and, in particular, the use of social media.

### Recommendations

We integrated the results of the review and the patient advocate’s viewpoint to identify practical guidelines on how to increase PPI in future epidemiological studies. We used the implementation of a digital cohort study as an exemplary case for testing and illustrating established guidelines for PPI [15]. In addition, we integrated in our recommendations the revised version of Guidance for Reporting Involvement of Patients and the Public (GRIPP2) checklist as an instrument to improve the quality of PPI reporting [16].

This study was based on a collaboration of patients and researchers. A patient advocate (RS), patient researchers (TS and TB), and researchers (GAA, CG, AF, VTT, PR, LH, and GF) were involved in the preparation of the manuscript (cowriting, editing, and critical review).

## Results

### Review of PPI Methods

**Figure 1:**
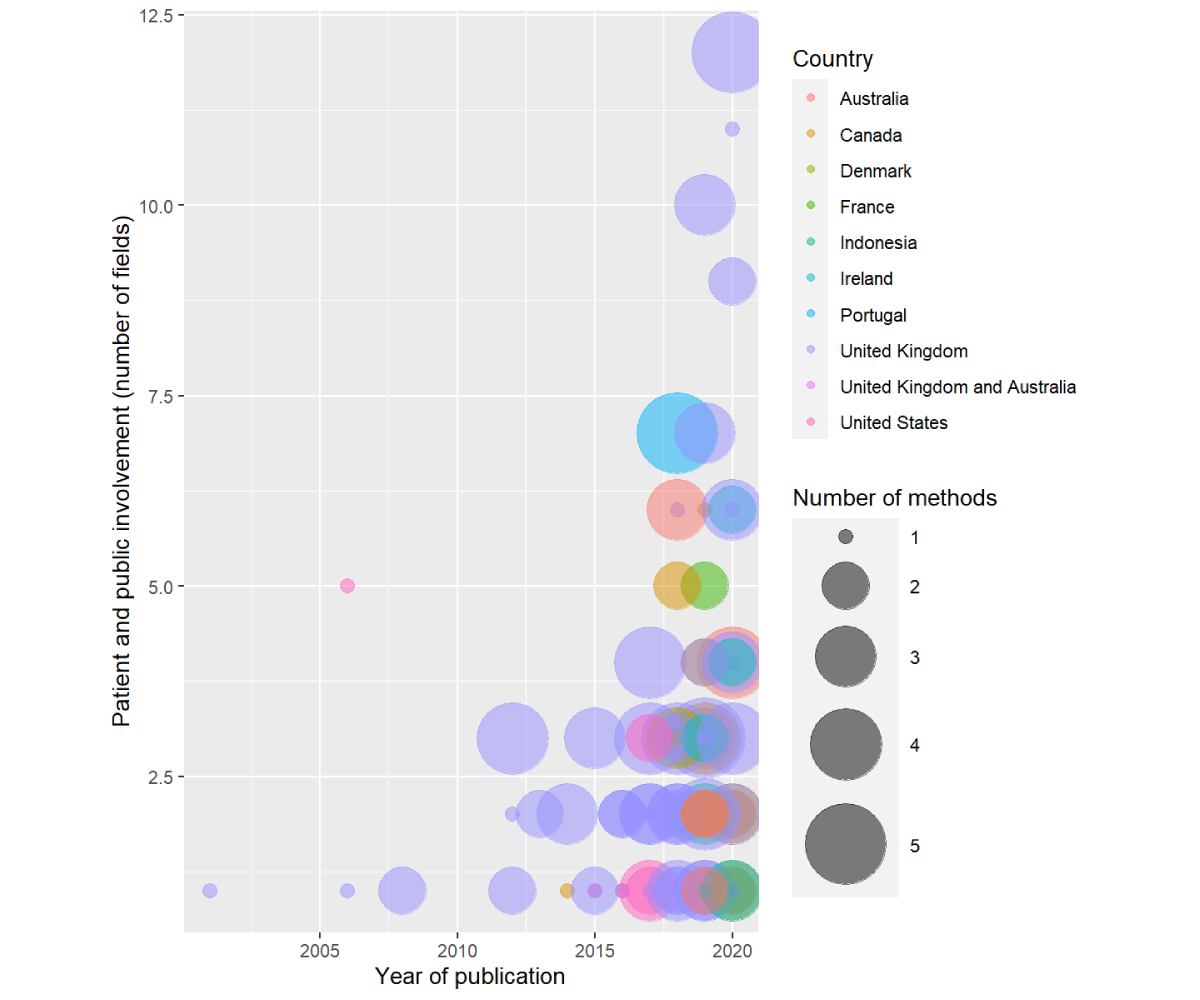
Number of fields or areas (ie, 1 field=involved in research ideas and 2 fields=research ideas and co-design) in which patients, carers, or the public were involved (y-axis); number of methods (circles); and countries (colors) where the studies were performed from 2000 to 2020 (x-axis). Patient and public involvement increases over time and at different stages of involvement. The size of each circle represents the number of methods used for patient and public involvement. Circles representing a combination of methods are very common in recently published studies. The most represented country is the United Kingdom.

We analyzed data from 97 studies published from 2000 to 2020. The studies were performed in 9 countries: the United Kingdom (58/97, 60%), Canada (13/97, 13%), the United States (8/97, 8%), Australia (6/97, 6%), Ireland (6/97, 6%), Denmark (2/97, 2%), France (1/97, 1%), Portugal (1/97, 1%), Indonesia (1/97, 1%), and the United Kingdom and Australia (1/97, 1%). The most frequent methods of PPI were workshops (47/97, 48%); surveys (33/97, 34%); meetings, events, or conferences (28/97, 29%); focus groups (25/97, 26%); interviews (23/97, 24%); consensus techniques (8/97, 8%); James Lind Alliance consensus technique (7/97, 7%); social media analysis (6/97, 6%); and experience-based co-design (3/97, 3%). Of the 97 studies, 34 (35%) used only 1 method, whereas 30 (31%), 22 (23%), 8 (8%), and 3 (3%) used 2, 3, 4, and 5 methods, respectively. The use of ≥3 methods together was observed from 2017 onwards (Figure 1 and Multimedia Appendix 1 [17-114]).

### Format of Involvement

#### Broad Definition of PPI Used

When considering methods of approaching PPI, 2 situations should be distinguished. The first situation is where patients or the public provide data on ideas or research priorities. The second situation is where patients or the public are actively involved in decisions about the research and perform or collaborate in the design, data analysis, data interpretation, writing, or diffusion of results. For this review, we used a broad definition of PPI that includes both situations: patients or the public involved in generating research ideas and patients or the public actively involved in the research cycle [115].

#### Workshops

We found that the most frequent method for approaching PPI was a workshop. Workshops are group activities where participants discuss a defined topic and decide actions. There are different ways of organizing a workshop. The first way is to organize it always in the same place and at the same hour. Morris et al [116] emphasized the importance of having enough space and time for the discussion. In addition, the number of participants by workshop or by discussion group may be limited. Mackintosh et al [17] proposed separate groups of users and service providers of 3-4 people. In contrast, Kelemen et al [18] organized full-day workshops with 20 to 25 participants of different origins and ages and divided into 2 groups randomly assigned. Patients prefer places with quiet rooms and parking lots, with meeting and meal times that allow them to participate and manage their medications [19]. An alternative to a workshop held at a specific location is to organize mobile workshops to facilitate the participation of people with busy schedules or mobility issues. Eccles et al [20] organized this type of workshop with a maximum of 10 participants and a short duration (30 minutes). These workshops were usually offered on the sites of existing organizations, which improved the success rate [20]. When face-to-face workshops are not possible, a workshop by videoconference can be organized [21]. Workshops can also be organized as consensus meetings [15,22], with a second-round workshop [117] or by applying other techniques to improve explicit involvement, such as experience-based co-design [23]. Workshops are usually analyzed with qualitative research methods such as thematic analysis [118] (Multimedia Appendix 1).

#### Surveys

Surveys aim to obtain information in an easy, individual, and feasible way. They can include open-ended questions. For example, a citizen science study asked participants the *magic wand* question (“If you had a magic wand, what would you change in your healthcare?”) [24]. Surveys can also include closed-ended questions. Little et al [25] performed a study to identify patients’ preferences for a patient-centered consultation using Likert scale questions that were previously tested and validated in a pilot study. To respond to a survey, patients and the public could be contacted in person [25], through a web-based survey [119], or by telephone [120]. Several studies included a second round of questions for improving ideas or establishing priorities [26,121].

#### Focus Groups

Focus groups are group activities, a form of qualitative research that includes people with certain similarities such as a therapeutic area or illness or demographic or socioeconomic group to discuss their opinions and beliefs about a topic familiar to the participants [27]. An advantage of focus groups is the potential to obtain multiple opinions and different understandings of the same question, which can enrich explanations for a certain issue. A disadvantage is that focus groups require a trained interviewer to conduct them, and interaction among participants can influence the outcome [122].

#### Meetings

Meetings are group activities where patients, carers, caregivers, and researchers participate in the project discussing and co-organizing activities for PPI [15]. They are complementary to workshops or focus groups and are also used to prepare these activities and to debrief methods or actions for PPI [28]. Meetings can be organized as festivals and public events, conferences [29], or web-based activities [30].

#### Semistructured Interviews

Semistructured interviews are a qualitative research method where the researcher discusses a defined topic individually and informally with the participant [123]. Using this method, nonresponse or direct behavioral observations, individual reflections on a specific research question, can be incorporated as outcomes; the advantage is that the participant is not influenced by other persons [124,125].

#### Consensus Techniques

Consensus techniques analyze a project’s chances of success by bringing together a group of experts at a workshop, meeting, or conference to discuss solutions and reach agreements [31]. James Lind Alliance is a consensus technique bringing clinicians, patients, carers, and the public together to achieve the convergence of opinions for establishing research priorities. The process starts with a web-based survey to collect research ideas from the public. Next, only unanswered research questions are selected. With a second web-based survey, the public prioritizes the selected questions. At the end of the process, the steering committee chooses the top 10 research priorities [126].

#### Persona-Scenario

*Persona-scenario* is a method for co-design in PPI where a fictitious user (persona) is created to communicate in a committed manner. Next, a scenario is proposed based on a story with an actor (the persona), a framework, a goal, actions to reach this goal, and obstacles. Participants can be asked to evaluate the extent to which they agree with the choice of the persona or what they would do if they were in the situation of the persona. This distancing—giving advice about someone else’s choice rather than answering for yourself—can help address sensitive themes [23,32].

#### Experience-Based Co-design

Experience-based co-design is a technique using narratives, usually video recorded, that allows patients and researchers to work in partnership to co-design new services and technology with the objective of improving the quality of health services. This technique has been applied with success for fostering PPI [33-36].

### Digital Methods to Promote PPI

Digital methods can be enablers of PPI, and the public can help modulate and develop digital technology [127]. Dedicated websites can enhance PPI not only by providing information, but also by organizing PPI itself. An example is the use of a webpage to organize a patient-led research hub. This is an initiative aimed at making patients and the public leaders of their own research projects. The researchers assist and support the patients and the public who proposed projects [37].

Crowdsourcing is a method by which many people are engaged on the web for a common goal such as obtaining new ideas and analyzing data. The main advantage is that it is possible to have a large number of contributors in a very short time [128]. However, because crowdsourcing participants are generally younger than participants from traditional nonweb-based procedures, these participants are not always representative of the target population [129].

Social media analysis is the use of digital data from social networks for epidemiological purposes. This information increases knowledge about epidemiological trends and may be very different from information obtained by traditional methods [130]. Social network data sources can be used, for example, to obtain information about the patient’s research priorities [38]. This often generates large quantities of data and can offer the opportunity to use special techniques such as natural language processing for the analysis [131].

Web-based platforms are internet services where the public and innovators meet and can be created for allowing coworking [132]. Vasilica et al [39] reported creating a web-based network to co-design a social media–based platform with the aim of generating information to improve disease outcomes [39]. In addition, web-based platforms through a web-based voting system have been used for establishing research priorities [133].

### Fields of Involvement

We found that many studies (60/97, 62%) involved patients and the public for generation of new ideas. Patients or the public contributed also in coproduction, co-design, or study scope (42/97, 43%) and in establishing research priorities (31/97, 32%). Other forms of PPI included participation in data analysis (25/97, 26%), as coauthors of a scientific article (17/97, 18%), as members of a steering committee or advisory group (16/97, 16%), reviewing or writing protocols (14/97, 14%), in the interpretation of results (12/97, 12%), in the dissemination of results and advocacy (11/97, 11%), in the data collection (9/97, 9%), in the development of the recruitment strategy (7/97, 7%), as a project manager (4/97, 4%), and being coinvestigator or having patient- or public-led projects (2/97, 2%). We found only 1 study where patients were involved in the co-design of mobile health tools (1/97, 1%). Finally, recent studies reported an early PPI in at least three different stages or fields of involvement and throughout the life of the research project (Table 1 and Figure 1).

**Table 1:** Fields of involvement in the included studies (N=97).

Type of involvement	Frequency, n (%)	References
Generation of new ideas	60 (62)	[18,20,22-27,30,32-36,38-82,134]
Coproduction, co-design, or study scope	42 (43)	[17,19,23,30,32-37,39,41,49,54,55,59,60,65-69,74,75,78,81,83-98]
Establishing research priorities	31 (32)	[24,26,29,30,34,35,41-43,46-48,53,54,57,60,62-64,66,72,75,77,81,83,89,90,99-102]
Participation in data analysis	25 (26)	[24,30,31,34,51,60,61,66,69-71,78,81,87,90,91,94,96,103-109]
As coauthors of a scientific article	17 (18)	[24,30,31,57,60,61,68,69,73,83,86,94-96,105,110,111]
As members of a steering committee or advisory group	16 (16)	[26,28,30,31,35,42,43,53,62,76,78,84,91,106,110,112]
Reviewing or writing protocols	14 (14)	[30,49,51,59,60,68,73,74,78,83,91,95,111,113]
Interpretation of results	12 (12)	[24,30,41,49,60,66,69,78,91,94,96,104]
Dissemination of results and advocacy	11 (11)	[30,39,41,49,57,60,70,78,86,94,104]
Data collection	9 (9)	[54,60,66,70,88,91,94,96,114]
Development of the recruitment strategy	7 (7)	[41,60,66,68,91,104,111]
Project management	4 (4)	[30,37,78,94]
Coinvestigator or having patient- or public-led projects	2 (2)	[37,94]
Co-design of mobile health tools	1 (1)	[135]
At least three different stages or fields of involvement and throughout the life of the research project	39 (40)	[24,26,28,30,31,34,35,39,41-43,49,51,53,54,57,59-61,66,68-70,73-75, 78,81,83,86,89-91,94-96,104,110,111]

To develop a successful PPI project, patients or the public and researchers must have, or develop, certain skills. For example, researchers need to become familiar with PPI as a research approach, know how to manage a PPI project, and how to deal with conflict. As for the patients and the public, they must understand the research process and develop capacities for management and conflict management. However, it is not mandatory that patients have specific vocational or educational training [136].

### PPI in Epidemiology: Trials and Cohort Studies

Web-based trials are more and more frequently described in the literature. Price et al [137] performed a systematic review of web-based trials and found that PPI was only reported in 24% (10/41) of the trials included in the review. Face-to-face meetings and email contact were the most common ways of interaction [137].

Taylor et al [97] performed a cohort study in patients with cancer that involved the patients in the creation and choice of a brand for the cohort. With a 1-day workshop, patients and researchers co-designed the brand. The results showed higher acceptance and retention of the study than expected. An ongoing cohort study used social media (Facebook) for PPI by creating a closed group of patients and families to bring new ideas to the project [80]. Meetings with a family advisory committee were organized regularly.

Morris et al [116], in the context of an epidemiological study, investigated PPI with surveys and postevent interviews and wrote recommendations. Before an event, they suggest sending a detailed document with the topics to be discussed. During the event, they suggest having enough space between tables to allow all participants to be heard, providing materials to facilitate note taking, taking a whole day for the meeting, and arranging a facilitator for each table. Finally, after the event, they recommend a follow-up by sending the notes to the participants [116].

### Barriers to PPI

Domecq et al [138] in a systematic review described some barriers to PPI. They highlighted 2 barriers: the excessive time taken for training activities and attendance and the risk of a tokenistic involvement. Another barrier reported in participants who were frail was frustration because of discontinuity in the involvement [139]. Barriers reported by researchers were concerns about the quality of research, ethical issues, lack of funding, failure of the PPI in the past, and not being convinced of the real need for PPI in the cohort [140]. Maccarthy et al [113] described communication issues as a key barrier to PPI. Researchers fear not being able to explain the project and not being able to engage patients and the public in the project; they also feel discomfort speaking about their experiences with patients and the public and fear having misunderstandings.

### Facilitators of PPI

Creating a safe and welcoming environment where each contributor feels empowered and confident facilitates PPI [94]. The coproduction process can give participants the self-confidence to take responsibility for the entire duration of a project [78]. In addition, an iterative process of PPI evaluation during the entire research cycle has been proposed to ensure success in PPI [113]. Mathie et al [141] reported that feedback for patients, when provided, motivated them to continue their collaboration with researchers. Concerns for well-being, trust, mutual respect, and flexibility in time and methods were facilitators of PPI [139]. Chambers et al [142] found key areas that may facilitate or hinder the development of PPI. These key areas were the following: good role definition, recognition of difficulties, integration through organizations, training, developing networking, considering different perspectives, improving communication, and recognizing the relevance of emotional impact. Finally, concerning digital interventions and PPI, O’Connor et al [143] recommend investing in raising awareness of the usefulness of digital tools, improving health literacy, and using optimal tool design.

### A Patient Advocate’s Viewpoint of PPI in Diabetes Research

When writing this review, it was natural to allow a potential representative of study participants to express how they see PPI so far in research and what they are expecting from researchers to increase the participation of people with diabetes in research (Textbox 1, written by RS).

**Involving people with diabetes in research: a patient advocate’s viewpoint.**

**Viewpoint of a patient advocate**

- On November 14 each year, World Diabetes Day is celebrated across the globe. The International Diabetes Federation with the World Health Organization created this awareness campaign in 1991 to respond to the growing numbers of people with diabetes worldwide. In 2006, World Diabetes Day was deemed an official United Nations Day with a special resolution, becoming only the second health condition so recognized.
- Why November 14? That date marks the birthday of Sir Frederick Banting, who, along with Charles Best, is credited with the discovery of insulin. The day is acknowledged by diabetes organizations, health care professionals, researchers, and governments. Most importantly, people with diabetes have embraced the day to acknowledge, commemorate, and also celebrate life with diabetes as we gratefully signpost the man whose research is responsible for our very lives.
- People with diabetes are interested in research. We know that the developments we see each and every year that advance how we live with diabetes are the result of research. We are interested in the different branches of research—clinical, educational, social, and behavioral—because we know better than anyone that living with diabetes is a multi-pronged existence that affects every part of our lives.
- However, despite how much we appreciate the work of researchers and how keen we are to learn more, sometimes it is difficult to engage with us and involve us as participants. Let us explore how we can address this gap and consider some changes that can be made to encourage people with diabetes to take more interest in research.

**Tell the story**

- The story of Banting and Best is folklore for those of us living with diabetes. It is a compelling story, but so are many other research tales. Unfortunately, the narrative is not always told especially effectively. It is difficult to make research sound relevant to people living each day with diabetes when research involves cells in a petri dish or stem cells in a temperature-controlled laboratory. What is the difference this work will make to our day-to-day lives?
- It is exhausting for us to hear how mice are cured of diabetes (once again), especially when we know that our cure is still as elusive as ever.
- However, these stories—the cells and the mice—are links in a long chain that lead to significant developments that do directly affect us. At the moment, explaining that seems lost in translation, and researchers need to think about how to decode in basic language how the work they are doing has the potential to make significant changes to the everyday life of people with diabetes and that participating in relevant research gets us to that goal.
- Even those researchers whose work is more practical based are not always especially successful in describing the impact of their work on those of us with diabetes. Plain language statements are a start, but looking for even more nuanced and targeted ways to communicate is important.

**Tell it in a tweet!**

- With 280 characters on offer, Twitter is the perfect platform for researchers to hone their short story–telling skills. Practice the elevator pitch of your research by narrowing down the key points and benefit to people with diabetes, and share it on the web to encourage interest. (Twitter threads allow for linking a number of tweets together, so if you need more than 280 characters, you can take a couple of tweets. But do keep it brief!)

**What is involved?**

- When recruiting people for your research, be very clear about what they will need to do. How much time is involved? Where will they need to go? Will there be any invasive procedures and how uncomfortable are they likely to be? (Be honest!)
- Follow-up is critically important. A complaint we hear from people participating in research is that once their involvement is over, they never again hear from the research team. This can be especially frustrating if people have invested a lot of time and energy in a trial. Regular updates through a newsletter or social media page keep people informed and linked in with your work. This is especially important if you are planning to recruit people for future phases of your study.

**Participants, not subjects #LanguageMatters**

- The words you use when communicating to, and about, people with diabetes are critically important. Refer to language position statements developed by diabetes organizations to ensure that your language is supportive, empowering, positive, and encouraging. We people with diabetes are more inclined to be involved if we see a study that treats us with respect.

**The 2 camps—and how to bridge them**

- There seem to be 2 main camps when it comes to diabetes research. Some believe that the focus should be primarily on finding a cure for diabetes. This seems to be especially prevalent in the type 1 diabetes space, with much of this thinking led by parents of children living with diabetes. In 1970, it was these parents who founded the leading diabetes research organization, the Juvenile Diabetes Foundation (renamed the Juvenile Diabetes Research Foundation), now known as JDRF. The original organization’s mission was very clear: to find a cure for diabetes. In recent years, however, the research funded by JDRF has branched out to include studies looking at improving management through technology and drugs.
- However, it is important to acknowledge the importance of research that looks at better management. Without this research, there would be no treatment for diabetes-related complications and we would not have technology such as home blood glucose meters, insulin pumps, continuous and flash glucose monitors, algorithms to automate insulin delivery...and we would still be using the same insulin from dogs that Banting and Best had used.
- Just as important is the growing body of work and researchers dedicated to researching the social, psychosocial, behavioral, and emotional aspects of living with diabetes. As anyone living with diabetes will tell you, this condition is never just about metrics. It is very much about our *headspace* and how we feel about living with diabetes.

**Involve us**

- When is the best time to start to involve people with diabetes in your research? It is probably already too late! Have you consulted us when you were establishing your study design? And back up a little more...is the research really something that is going to be of interest or benefit to people with diabetes. Is the problem you are looking to solve really a problem for us?

**Patient advisory committees**

- Many research bodies now require patient advisory committees to be established as part of the overall study. Done well, these groups can provide invaluable input for projects. Done badly, they are nothing more than a tick-the-box exercise. Ensure that there is funding available for travel, accommodation, and other expenses. Be clear about what you expect the patient advisory committee members to do and which aspects of the project they will be involved in. Remember that patient advisory committee members will most likely be volunteering their time. Their expertise and time should be reimbursed by honoraria or hourly payments.

**Not enough money in the pot**

- Research dollars are never enough, and each year, there are more researchers contending for elusive grants. When the results from grant rounds are shared, it seems that diabetes is repeatedly the *poor cousin* of health conditions, regularly being awarded significantly less money (with fewer successful grants) than conditions such as cancer and cardiovascular disease. In recent grant announcements from the National Health and Medical Research Council in Australia, only 16 diabetes grants worth Aus $13.5 million (US $9.6 million) were awarded compared with 69 grants worth Aus $52.9 million (US $37.8 million) awarded for cancer research.
- People with diabetes can help to advance the cause of diabetes grant applications by telling their stories. Perhaps one of the reasons that diabetes receives comparatively little of the research bucket is because we have not been all that successful in telling our stories. Instead, we have created a false image of diabetes as a hugely preventable, self-inflicted condition, resulting in government and other research bodies considering diabetes a less worthy condition to fund.
- Researchers are encouraged to work closely with people with diabetes to help tell the story of why their own research is important and how it has the potential to help in the lives of people affected by, or at risk for, diabetes. Humanizing the story is important—all too often, diabetes is presented in the media as a headless overweight body, which only adds to the stigma and image problem of the condition.

**The story of hope**

- Research is selling an important feature: hope. People with diabetes trade on hope; we look for it in research because we know that is what holds the key to improving outcomes, reducing burden, and making our diabetes lives easier. We want to be part of those discoveries that promise a better life, and we want to be involved in your research that will help us get there.

### Illustration of PPI in a Digital Cohort Study

Digital cohort studies are longitudinal studies in which the data come either totally (e-cohort) or partially (hybrid: e-cohort and traditional cohort) from digital sources. Modern cohort studies increasingly incorporate digital tools such as data generated on the web and connected devices that allow much wider use of data generated for multiple projects [9].

We elaborated a strategy of PPI for a digital cohort study. Tables 2 and 3 show recommendations for PPI at all stages of research. This participation was defined in 2 categories: *Recommended participation activities*, in which patients and the public participate more passively, helping to generate research ideas and prioritize those ideas by participating in surveys, and *Recommended involvement activities*, in which patients actively participate in collaborative work with researchers on an equal footing. Examples of these activities are events, meetings, workshops, and focus groups. The chosen strategies for the digital cohort study are detailed in 32 actions in total for participation as well as participation and involvement, corresponding to the different steps of the research process, and are based on the current recommendations of Involve [7], our review results, and the viewpoint of a patient advocate (Textbox 1). In addition, for the realization of certain activities, we suggest a recommended duration of the activity based on our review of the literature and the point of view of the patient advocate.

**Table 2:** Recommendations for the promotion of patient and public involvement (PPI) projects: concrete examples for a digital cohort study. Steps to be taken before starting the cohort study.

Stages (Involve list)	Suggested actions	
	Recommended participation activities	Recommended involvement activities
Identifying and prioritizing research axes	Web-based survey through social media: identification of research questions. Duration of the activity: 15 minutes	Videoconference meeting: establishing an international scientific steering committee with researchers and patients as members. Duration of the activity: 1 hour. Preparation: read agenda that should be sent 1 day before the meeting Meeting: identification of, and invitation to, a group of patients interested to be involved as patient partners (eg, through patient associations). Duration of the activity: 3 hours. Preparation: not needed. Venue: local patient association Videoconference meeting and use of web-based collaboration tools: cowriting PPI plan for the cohort and submission to an ethics committee. Duration of the activity: 2 hours. Preparation: read the proposal draft sent and written by researchers 1 week before
Designing	Web-based survey through smartphone app: identification of research questions, web-based survey with open-ended questions. Duration of the activity: 10 minutes Web-based survey: ranking research questions (through smartphone app, web-based survey using persona-scenario technique) with closed-ended questions. Duration of the activity: 15 minutes	Web-based or mobile workshops: coproduction by giving feedback on study design and chosen questionnaires and research tools (such as mock-ups of app, user experience and user interface). Duration of the activity: 3 hours Web-based training: language matters. Searching, choosing, and checking the most appropriate use of language for communication with the public and patients. Duration of the activity: 2 hours Mobile focus group and survey (Multimedia Appendices 2 and 3): assessment of the beta version of smartphone app and flyers, as well as assessment of the wording and visual of the website, flyer, study objectives, and PPI expectations. Duration of the activity: 4 hours. Meeting place: comfortable, with catering and parking lot available
Drafting grant protocol	—^a^	Web-based meeting: cowriting study protocol, involvement of patient associations as partners in grants. Duration of the activity: 1 hour. Preparation: read agenda that should be sent 1 day before the meeting
Testing and scaling up	Surveys through smartphone app: testing of pilot study by limited number of potential study participants. Duration of the activity: 30 minutes	Web-based meeting: co-designing pilot study on smartphone app. Duration of the activity: 1 hour. Preparation: read agenda that should be sent 1 day before the meeting Web-based meeting: co-design of generalization phase and recruitment. Duration of the activity: 1 hour. Preparation: read agenda that should be sent 1 day before the meeting Web-based meeting: advertise through social media and press for patients and the public to participate in the study

^a^No specific recommendations.

**Table 3:** Recommendations for the promotion of patient and public involvement (PPI) projects: concrete examples for a digital cohort study. Steps to be taken during the cohort study.

Stages (Involve list)	Suggested actions	
	Recommended participation activities	Recommended involvement activities
Analyzing and interpreting	—^a^	Web-based meeting using web-based collaboration tools: cowriting of annual reports Webpage, web-based workshops, meetings, and web-based collaboration tools: coproduction of research projects through a patient-led research hub. Webpage with a dedicated section for submission of projects by patient. Projects assessed by the scientific steering committee Web-based workshops, meetings, and web-based collaboration tools: data analysis and interpretation of results Meetings and web-based collaboration tools: writing of manuscripts cowritten by scientists and patients
Disseminating	—	Social media: dissemination of publications coauthored by scientists and patients Web-based meetings, workshops, and web-based collaboration tools: participation at conferences as author or coauthor Focus groups and workshops: communication of research results (plain language, infographic, and dissemination)
Implementing	—	Web-based meetings and workshops: implementation of some results from the study at hospitals and consultations facilitated by patients
Monitoring and evaluating	Smartphone app: improving participants’ retention by reminders Email newsletter and social media announcement: follow-up of the project by researchers (once a month) One-day general public event: follow-up of the project by researchers (once a year) Web-based survey through social media and smartphone app: monitoring evolution of the research protocol (adding or deleting research questions)	Web-based meetings: checking of data collection and data quality by patients Web-based meetings and web-based collaboration tools: review of research projects by patient representatives Social media: improving participants’ retention by involved patients One-day general public event: follow-up of the project by patients and public (once a year). Remuneration or facilities for attending should be budgeted for members of the scientific committee Workshop: evaluation of PPI by researcher and contributors (use GRIPP2^b^ checklist). Duration of the activity: 1 day. Meeting place: comfortable with catering and available parking places Meetings and workshops: Monitoring evolution of the research protocol (adding or deleting research questions) by scientific steering committee (scientists and patient members)

^a^No specific recommendations.

^b^GRIPP2: revised version of Guidance for Reporting Involvement of Patients and the Public.

The advantage of a digital cohort study is that digital tools can be used to promote PPI at each stage as a primary method or as a complementary or alternative method. A digital cohort can facilitate PPI, allowing participation from remote locations, using a smartphone app with web-based questionnaires, organizing most of the meetings through videoconferences, and using web-based tools for coworking. However, we think that face-to-face activities are also recommended and these 2 approaches may be complementary.

The recommendations are as follows:

- Recommendation 1: Identify patients and the public who might be interested in participating as members of the patient advisory steering committee. This contact can be achieved through social media and patients’ organizations. Use digital tools to identify people. For example, contact organizations that are active on forums or social media.
- Recommendation 2: Write a PPI protocol in the digital cohort protocol describing all planned activities and include funding for patients. The patients who are part of the steering committee should be actively involved in this activity.
- Recommendation 3: Identify patients and the public who might be interested in participating in focus groups, semistructured interviews, or workshops by being involved actively in the design of the app, website, research ideas, and project evaluation.
- Recommendation 4: Organize focus groups as an important activity to obtain information about how PPI can be integrated in a digital cohort study. We present an example guide for this activity in Multimedia Appendix 2. A passionate and enthusiast moderator is needed. Sometimes it is not possible to find a place and a time that works for everyone. In such cases, mobile or web-based focus groups can be organized.
- Recommendation 5: Involve patients in the design of the smartphone app or other digital tools. For working on the design of a smartphone app, we encourage the use of an evaluation grid to assess the app with direct observation of how patients and the public use it. We present an example of an evaluation grid in Multimedia Appendix 3. Patients and the public can also assess a smartphone app using a validated scale. We suggest the application of the user version of the Mobile Application Rating Scale [144].
- Recommendation 6: Involve patients in the generation of new research ideas and research priorities. Using a smartphone app or through a website, we recommend that during the study, participants be invited to propose research questions at any time and as often as they wish. The patient advisory steering committee is involved in all the discussions about research priorities and follow-up of the project.
- Recommendation 7: Give feedback to patients and the public through web-based newsletters and social media. Evaluate regularly satisfaction with PPI. To assess the impact of PPI among active participants and to define roles and changes in the project, we advise that annual workshops should be organized. At these annual workshops, PPI can also be assessed by contributors (researchers, patients, and the public) using the GRIPP2 checklist [16].
- Recommendation 8: Establish a patient-researcher partnership. On the cohort’s website and in the smartphone app, we encourage the creation of a *patient- or public-led research hub* space. This space can receive applications from patients or the public to develop research projects. The scientific steering committee assesses applications concerning scientific content and feasibility. The research team helps patients and the public to carry out their own projects by providing methodological support.
- Recommendation 9: Finally, we recommend inviting patient advocates, patients, or caregivers to become *study ambassadors*. They are volunteers actively involved in the study and social networks. The role of the study ambassadors will be to actively disseminate the results of the study and invite their networks to join the project.

**Figure 2:**
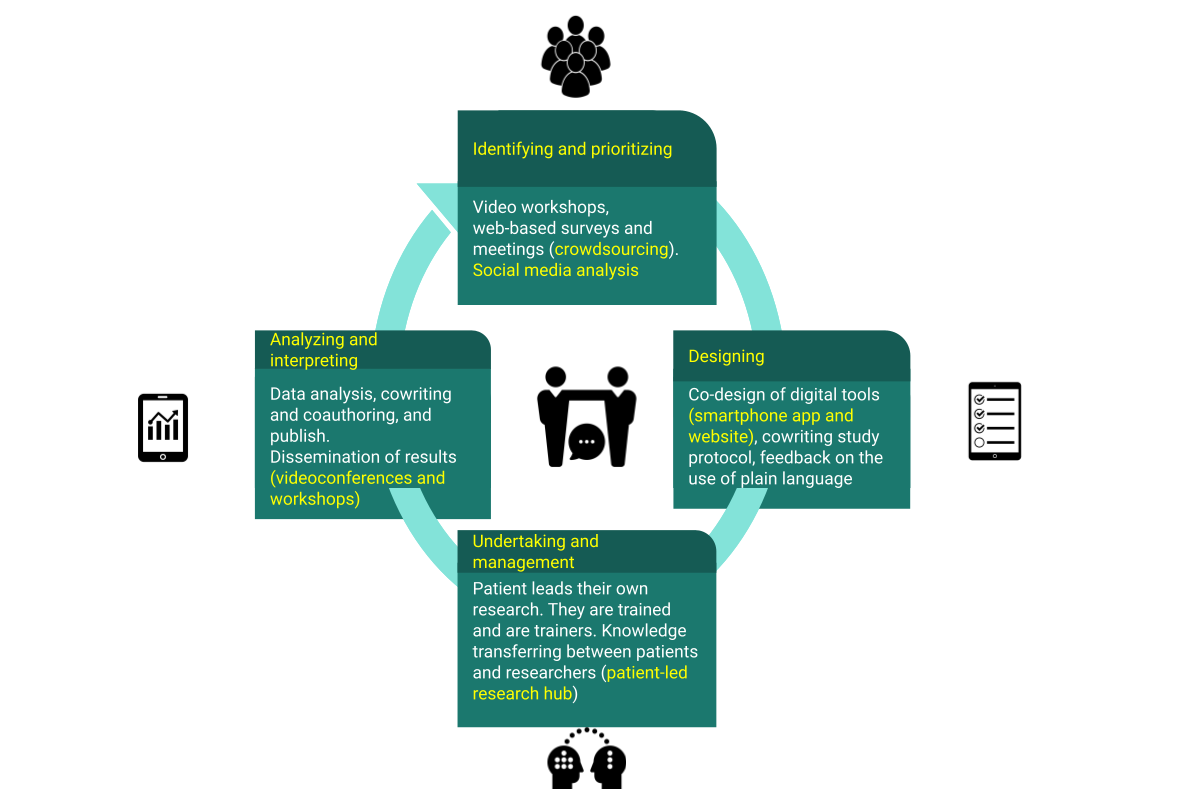
Patient and public involvement in the research cycle of a digital cohort study. Digital tools are integrated at each stage of the research cycle, and some examples of digital tools are shown in the figure.

Figure 2 shows our vision of integrating PPI in a digital cohort study in the whole research cycle.

## Discussion

### Principal Findings

We observed that the most popular methods for PPI are workshops, focus groups, interviews, and surveys. The appropriate method must be carefully chosen to fit the research objective. For example, workshops and focus groups can be a rich source of information in the prestudy phase; they can be adapted to the context of the participants; and they allow direct observation of, and interaction among, participants. The downside is that they are highly dependent on the capacity of the moderators.

We have also described digital tools to collect information from the patients and the public for research, such as social media listening and a particular web-based crowdsourcing survey with the *magic wand*–like question [24]. We think that digital tools may not only facilitate patient engagement in research, but can also stimulate a continuous and long-term participation and involvement of patients and the public.

Overall, none of these methods can address all relevant questions for scientists and patients or the public related to a research project. Therefore, we recommend a mix of methods to obtain optimal and meaningful information for the research project at stake. Different methods can be applied at different times of the project depending on the objective. Regarding the timing of introducing PPI, we recommend that PPI be included from the beginning (eg, co-design and research plan) to the end (publication and dissemination), followed by a PPI assessment.

We suggest pragmatic steps to integrate PPI in future epidemiological studies. These recommendations are as generic as possible but may not be applicable in all cases. They are based on current guidelines for PPI with concrete examples for a digital cohort study [14,15]. These recommendations should be understood as modular, meaning that they must be adapted to the study design, population, available budget, duration of the project, and local context. For PPI report and assessment, we recommend using the GRIPP2 checklist, a dedicated PPI reporting checklist [16].

### Comparison With Prior Work

Domecq et al [138], in a systematic review, identified barriers to, and facilitators of, PPI. They found no evidence indicating which method was best for PPI. In our review, we found that a combination of methods is more common with greater involvement (patients included in many areas or across the research cycle). We integrated the barriers and facilitators described by Domecq et al [138] in our recommendations.

Liabo et al [145] performed a systematic review of good PPI practices. They compared the results of the systematic review with 3 involvement groups and found that the priorities were similar. However, the involvement groups found additional values that were not described in the literature, such as the enthusiasm of the participants and the choice of welcoming venues for the meetings. We integrated these reflections in our recommendations.

Nunn et al [146] reviewed reports of 96 human genomic research projects and found that only 33% (32/96) declared PPI. From these, most of the PPI activities were organized in formal groups (20/32, 63%), with 22% (7/32) using web-based tools (website, social media, and web-based communities). We found similar results with social media reporting in 6% (6/97) of the studies with PPI. We think that there is room for more PPI using digital tools.

Miah et al [147] conducted a scoping review of PPI in dementia research and found 19 studies from the United Kingdom and 1 from the Netherlands. Biddle et al [13] found an uneven distribution of PPI in Europe. They attributed this to a lack of infrastructure, support, and guidance. An example of support is that research-funding institutions in the United Kingdom require PPI in project applications. However, funding agencies in many countries do not have this requirement. We found similar results with most of the studies on PPI from the United Kingdom. We believe that PPI is still underreported or not performed in many countries.

Few epidemiological or clinical studies report PPI in the research process [11,146]. Studies that include patients and the public most often involve them only at the stage of idea generation, but not in the whole research cycle. This suggests that PPI is symbolically added or very limited.

Individual interviews are useful for tackling sensitive questions because participants do not feel dominated or influenced by the opinion of other participants. Surveys (web-based, telephone-based, or paper-based) are a pragmatic method of obtaining large-scale information from many people quickly. Differently, a survey can be very useful for generating new ideas and for refining and improving them when a new survey containing the generated ideas is launched for another sample of people.

PPI is a *project in the project*, creating its own challenges such as completing appropriate regulatory tasks and obtaining approval from an ethics committee. In the informed consent, the nature of participation must be specified, with clear and fair terms and conditions. This includes whether remuneration, cost coverage for the patient organization, and travel costs of patients or the public are provided and whether there are other benefits for the patients, such as nonfinancial compensation for the time allocated to project participation [14].

Several reasons for a lack of, or delay in, PPI have been mentioned in the literature. Some researchers do not include patients or the public in the research, arguing that the patient or public point of view is too subjective [148]. Furthermore, researchers may consider PPI only as a requirement of funding agencies for the project to be approved; therefore, PPI arrives late in the process and is treated as an afterthought [149]. We believe that obstacles can be overcome when they are identified and taken into account in the plan and recommendations.

When PPI is considered, there is the risk of selective PPI, which means that only those within the community who agreed with the research objectives are included. In addition, this may have the risk of hearing only the opinion of the most active patients or public, which may not reflect the opinion of most of the other patients and can therefore lead to research designs that still do not research the questions that interest most patients. We recommend nonselective PPI, meaning that the aim should be to involve a diverse selection of participants in the PPI process, including patient organizations, patient advocates who are legitimately speaking on behalf of a patient community, and individual patients, to ensure that the opinions and views of the participants are representative of most patients with the same condition [47].

Researchers should make a greater effort to minimize the burden of participation for patients and maximize the benefits for participants at the same time. A lack of participation is more likely to occur when there is poor or 1-way communication, resulting in poorly organized protocols and demanding follow-ups that lead to noninformed, noninvolved, and nonmotivated participants. Nevertheless, when done properly, patients can associate their participation with feelings such as usefulness, empowerment, and consideration [150].

### Limitations

This study includes certain limitations. Our search was limited to studies in English. In addition, a part of what is happening in PPI is described in the gray literature and has therefore been excluded from the review. Our review may give the impression that early involvement in setting research priorities is the norm, but this may not be true because of a potential publication bias. For example, the results of studies with PPI are often not intended to show improvements in efficacy in clinical studies; therefore, they are less likely to be accepted by publishers for publication or publication may be delayed because of negative or statistically insignificant results.

### Conclusions

There are, and rightly so, many expectations on the part of patients and the public to be actively involved in research and not only by providing data, but also as research partners. PPI can contribute to patient empowerment by increasing disease awareness and according recognition as actors of their own condition [151]. However, PPI is uneven among countries and research institutions, and even now many patients and the public are not yet involved in research [13] and ignore or do not have access to research protocols [152]. Digital tools such as websites, social media, and connected devices have been increasingly incorporated into cohort studies and could be leveraged to increase PPI [153]. Digital tools can facilitate PPI by providing an opportunity for remote access and therefore easier participation. In addition, digital tools can facilitate PPI by enabling feedback and interaction between researchers and patient collaborators [154].

PPI can be a powerful approach to increase the relevance of research projects. We have shown that PPI must be planned in the initial phases of the development of a new epidemiological study and then be considered throughout the life of the research project. Combining different approaches of PPI seems to be the most effective strategy for improving the quality of research.

Some techniques such as *persona-scenario* are very powerful for idea generation and can be combined with digital tools. In addition, web-based surveys are easy to implement and allow involving many participants (crowdsourcing).

Digital methods such as social media listening or web-based *magic wand*–like questions can also offer useful complementary channels of interaction and help to identify key information such as research gaps at large scale with a limited cost directly from the target population. As such, we recommend that these methods be also integrated in the PPI process. With the example of a new digital cohort study, we offer practical guidelines to implement and run a patient- or public-centered research study. We therefore encourage investigators to rely on our practical recommendations to increase PPI in future epidemiological studies.

## Appendix

Multimedia Appendix 1 Methods and fields of patient and public involvement.

Multimedia Appendix 2 A focus group guide.

Multimedia Appendix 3 Grid for assessment of a smartphone app.

## Abbreviations

GRIPP2: revised version of Guidance for Reporting Involvement of Patients and the Public
PPI: patient and public involvement
