# Supplementary material for: Methods to Generate Innovative Research Ideas and Improve Patient and Public Involvement in Modern Epidemiological Research: Review, Patient Viewpoint, and Guidelines for Implementation of a Digital Cohort Study
Source: J Med Internet Res. 2021 Dec 23;23(12):e25743. doi: 10.2196/25743 (PMC8738987; doi:10.2196/25743)
Supplement: Multimedia Appendix 1 [file jmir_v23i12e25743_app1.docx]

## Multimedia Appendix 1. Methods and field of patient and public involvement

| **Author, year, reference** | **Country** | **Title** | **Method** | **Field of PPI** |
| --- | --- | --- | --- | --- |
| Bayliss, 2016 [1] | United Kingdom | Patient involvement in a qualitative meta-synthesis: lessons learnt | Survey and focus group | Data analysis. |
| Bell, 2019[2] | Canada | Co-building a patient-oriented research curriculum in Canada | Focus group | Co-design a research curriculum |
| Berwick, 2017[3] | United States | Breaking the Rules for Better Care | Surveys, interviews and meetings | Ideas for better care |
| Best, 2017[4] | United Kingdom | Network methods to support user involvement in qualitative data analyses: an introduction to Participatory Theme Elicitation | Focus group and meeting | Data analysis |
| Bethell, 2019[5] | Canada | The Canadian Frailty Priority Setting Partnership: Research Priorities for Older Adults Living with Frailty | James Lind Alliance method | Research ideas, steering committee |
| Birnie, 2019[6] | Canada | Partnering For Pain: a Priority Setting Partnership to identify patient-oriented research priorities for pediatric chronic pain in Canada | James Lind Alliance method | Research ideas, steering committee |
| Boddy, 2017[7] | United Kingdom | Does funded research reflect the priorities of people living with type 1 diabetes? A secondary analysis of research questions | Workshop | Research ideas |
| Boney, 2015[8] | United Kingdom | Identifying research priorities in anaesthesia and perioperative care: final report of the joint National Institute of Academic Anaesthesia/James Lind Alliance Research Priority Setting Partnership | James Lind Alliance method | Research ideas, steering committee |
| Brainard, 2017[9] | United Kingdom | Forced migrants involved in setting the agenda and designing research to reduce impacts of complex emergencies: combining Swarm with patient and public involvement | Interviews, surveys, focus groups and meetings | Research ideas, priorities, co-design, data collection |
| Brett, 2020[10] | Australia | The co-design of timely and meaningful information needed to enhance social participation in community aged care services: Think tank proceedings | Meetings and Delphi consensus. | Research ideas and co-design |
| Brooks, 2019[11] | Indonesia | Evaluating the acceptability of a co-produced and co-delivered mental health public engagement festival: Mental Health Matters, Jakarta, Indonesia | Workshop, survey and meeting | Research ideas and priorities |
| Brown, 2006[12] | United Kingdom | Discovering the research priorities of people with diabetes in a multicultural community: a focus group study | Focus group | Research ideas |
| Buffel, 2018[13] | United Kingdom | Social research and co-production with older people: Developing age friendly communities | Workshops and interviews | Co-design, data collection |
| Carr, 2019[14] | Canada | Co-design of a patient experience survey for arthritis central intake: an example of meaningful patient engagement in healthcare design | Meetings | Co-design, research ideas and priorities |
| Casanova, 2020[15] | United Kingdom | The impact of active research involvement of young children in the design of a new stereo test | Meeting and consensus activity | Research ideas |
| Cheraghi-Sohi, 2008[16] | United Kingdom | What patients want from primary care consultations: a discrete choice experiment to identify patients' priorities | Survey | Establishing priorities |
| Costello, 2020[17] | Ireland | Laying the groundwork: Building relationships for public and patient involvement in pre-clinical paediatric research | Workshop and meeting | All stages of the research cycle |
| Coultas, 2019[18] | United Kingdom | Patient and public involvement in priority-setting decisions in England's Transforming NHS: An interview study with Clinical Commissioning Groups in South London sustainability transformation partnerships | Semi-structured interviews and voting | Research ideas |
| Coupe, 2019[19] | United Kingdom | Patient and public involvement in doctoral research: Impact, resources and recommendations | Workshops, interviews, meetings and social media | Data analysis, data collection, study protocol, research ideas |
| Dahm, 2019[20] | Australia | Interaction and innovation: practical strategies for inclusive consumer- driven research in health services | Workshop | Co-design, data analysis priorities |
| Dawson, 2020[21] | United Kingdom | Patient and public involvement in doctoral research: reflections and experiences of the PPI contributors and researcher | Meetings | All stages of the research cycle |
| Dewa, 2020[22] | United Kingdom | Reflections, impact and recommendations of a co-produced qualitative study with young people who have experience of mental health difficulties | Workshop, interview, meeting, social media, and consensus techniques | All stages of the research cycle |
| Dias, 2018[23] | Portugal | Implementation Process and Impacts of a Participatory HIV Research Project with Key Populations | Workshop, interview, meeting and survey | All stages of the research cycle |
| Eccles, 2018[24] | United Kingdom | Patient and public involvement mobile workshops - convenient involvement for the un-usual suspects | Workshop | Research ideas |
| Evans, 2019[25] | United Kingdom | A co-produced method to involve service users in research: the SUCCESS mode | Workshop | Co-design design of the methodology of the model |
| Finer, 2018[26] | United Kingdom | Setting the top 10 research priorities to improve the health of people with Type 2 diabetes: a Diabetes UK–James Lind Alliance Priority Setting Partnership | James Lind Alliance method | Research ideas and prioritisation |
| Fonseka, 2020[27] | Canada | Collaborating with individuals with lived experience to adapt CANMAT clinical depression guidelines into a patient treatment guide: The CHOICE-D co-design process | Meeting and workshop | Co-design and co-write  the project |
| Frith, 2019[28] | United Kingdom | Role of public involvement in the Royal College of Physicians’ Future Hospitals healthcare improvement programme: an evaluation | Survey and focus group | Co-design |
| Frost, 2018[29] | United Kingdom | Patient involvement in qualitative data analysis in a trial of a patient-centred intervention: Reconciling lay knowledge and scientific method | Workshop | Generation research ideas and data analysis |
| Gadsby, 2012[30] | United Kingdom | Setting research priorities for Type 1 diabetes | James Lind Alliance method | Generation research ideas and data analysis |
| Garfield, 2015[31] | United Kingdom | Patient and public involvement in data collection for health services research: a descriptive study | Interview | Data collection |
| Garfield, 2016[32] | United Kingdom | Lay involvement in the analysis of qualitative data in health services research: a descriptive study | Interview | Data analysis |
| Ghisoni, 2017[33] | United Kingdom | Priority setting in research: user led mental health research | Workshop, voting questions,  nominal technique | Research ideas and priorities |
| Gillard, 2012[34] | United Kingdom | Patient and Public Involvement in the Coproduction of Knowledge: Reflection on the Analysis of Qualitative Data in a Mental Health Study | Workshop | Co-design and research ideas |
| Grant, 2020[35] | United Kingdom | Informing the development of an E-platform for monitoring wellbeing in schools: involving young people in a co-design process | Workshop | Research ideas and priorities |
| Gregg, 2019[36] | United States | A Novel Collaborative Approach to Building Better Clinical Trials: New Insights From a Patient Engagement Workshop to Propel Patient-Centricity Forward | Workshop | Research ideas and priorities |
| Grundy, 2019[37] | United Kingdom | Public involvement in health outcomes research: lessons learnt from the development of the recovering quality of life (ReQoL) measures | Interview, focus group and meeting | Research ideas and priorities, data analysis and interpretation, recruitment and data collection |
| Hatton, 2020[38] | United Kingdom | Innovative solutions to enhance safe and green environments for ageing well using co-design through patient and public involvement | Workshop | Research ideas and priorities, co-design and data analysis |
| Higgins, 2015[39] | United States | Medicare and Medicaid Users Speak Out About Their Health Care: The Real, the Ideal, and How to Get There | Focus group | Ideas for better care |
| Howe, 2017[40] | United States | Learning to work together - lessons from a reflective analysis of a research project on public involvement | Meetings and workshop | Advisory group (giving feedback for improving research) |
| Hyatt, 2020[41] | Australia | Co-design and development of online video resources about immunotherapy with patients and their family | Workshop, interview, video, experience-based co-design | Research ideas and priorities, co-design and steering committee |
| Irving, 2018[42] | United Kingdom | A coproduced patient and public event: An approach to developing and prioritizing ambulance performance measures | Workshop, meeting voting questions | Research ideas and co-design |
| Jennings, 2018[43] | United Kingdom | Best practice framework for Patient and Public Involvement (PPI) in collaborative data analysis of qualitative mental health research: methodology development and refinement | Meeting | Data-analysis |
| Jilka, 2019[44] | United Kingdom | Exploring patients' and carers' views about the clinical use of ketamine to inform policy and practical decisions: mixed-methods study | Survey and focus group | Data analysis |
| Jørgensen, 2018[45] | Denmark | User involvement in a Danish project on the empowerment of cancer patients - experiences and early recommendations for further practice | Interviews, workshops and focus group. | Reviewing protocol, data analysis , questionnaire development, co-writing and co authoring. |
| Kalot, 2019[46] | United States | An international survey to inform priorities for new guidelines on von Willebrand disease | Survey | Identification of priorities |
| Kelemen, 2018[47] | United Kingdom | Cultural animation in health research: An innovative methodology for patient and public involvement and engagement | Workshop and arts-based knowledge translation | Research ideas |
| King, 2019[48] | United Kingdom | Bringing together coproduction and community participatory research approaches: Using first person reflective narrative to explore coproduction and community involvement in mental health research | Interview, survey and focus groups | All stages of the research cycle |
| Knowles, 2018[49] | United Kingdom | Empowering people to help speak up about safety in primary care: Using codesign to involve patients and professionals in developing new interventions for patients with multimorbidity | Workshop | Ideas and co-design for intervention techniques |
| Kuluski, 2020[50] | Canada | An alternate level of care plan: Co-designing components of an intervention with patients, caregivers and providers to address delayed hospital discharge challenges | Focus group and patient's organisation, | Co-design and steering committee |
| Lee, 2019[51] | United Kingdom | Getting underneath the skin: A community engagement event for optimal vitamin D status in an 'easily overlooked' group | Workshop | Co-design, delivery, dissemination and evaluation |
| Leslie, 2019[52] | Canada | Recruitment of caregivers into health services research: lessons from a user centred design study | Focus group and nominal technique | Co-design, review protocol and co-author. |
| Little, 2001[53] | United Kingdom | Preferences of patients for patient centred approach to consultation in primary care: observational study | Survey | Research ideas |
| Locock, 2014[54] | United Kingdom | Using a national archive of patient experience narratives to promote local patient-centered quality improvement: an ethnographic process evaluation of ‘accelerated’experience-based co-design | Workshop and interviews | Research ideas/co-design |
| Locock, 2019[55] | United Kingdom | Involving service users in the qualitative analysis of patient narratives to support healthcare quality improvement | Workshop | Co-design and data-analysis |
| Maccarthy, 2019[56] | Ireland | Facilitating public and patient involvement in basic and preclinical health research | Survey | Reviewing protocol |
| Mackintosh, 2018[57] | United Kingdom | Employing the arts for knowledge production and translation: Visualizing new possibilities for women speaking up about safety concerns in maternity | Workshop | Co-design |
| Madden, 2020 [58] | United Kingdom | Producing co-production: Reflections on the development of a complex intervention | Workshop | Grant application, co-design interview and workshop, feasibility study, draft project |
| Mader, 2018[59] | United Kingdom | Inverting the patient involvement paradigm: defining patient led research | Patient lead research hub | Design of the project and study management |
| Mann, 2018[60] | United Kingdom | Reporting and appraising the context, process and impact of PPI on contributors, researchers and the trial during a randomised controlled trial - the 3D study | Meeting | Research ideas, analysis and writing |
| Mathie, 2020[61] | United Kingdom | The role of patient and public involvement leads in facilitating feedback: "invisible work" | Survey, interviews, focus group | Research ideas, data collection, analysis to dissemination |
| McCarron, 2019[62] | Canada | Understanding the motivations of patients: A co-designed project to understand the factors behind patient engagement | Survey | Patient co‐investigators and a researcher co‐designed and conducted this study |
| McCarron, 2020[63] | Canada | A co-designed framework to support and sustain patient and family engagement in health-care decision making | Interviews, survey, workshop | Co-investigators |
| Mulvale, 2020[64] | Canada | Finding harmony within dissonance: Engaging patients, family/caregivers and service providers in research to fundamentally restructure relationships through integrative dynamics | Focus groups and a co-design event | Co-design |
| Murta, 2019[65] | United Kingdom | The first UK national blepharospasm patient and public involvement day; identifying priorities | Survey, interactive voting, panel discussions | Research ideas |
| Neville, 2014[66] | Canada | The needs of persons with lupus and health care providers: a qualitative study aimed toward the development of the Lupus Interactive Navigator™ | Focus group | Research ideas |
| Ni Shé, 2020[67] | Ireland | Minding the gap: identifying values to enable public and patient involvement at the pre-commencement stage of research projects | Workshop | Draft and feedback of values statement, consensus discussion and co-author |
| Nissen, 2018[68] | Denmark | Patient involvement in the development of a psychosocial cancer rehabilitation intervention: evaluation of a shared working group with patients and researchers | Meetings | Co-design |
| O’Donnell, 2019[69] | Ireland | Enabling public, patient and practitioner involvement in co-designing frailty pathways in the acute care setting | Consensus building workshops | Co-design |
| O'hara, 2017[70] | United Kingdom | Strength in Numbers: an international consensus conference to develop a novel approach to care delivery for young adults with type 1 diabetes, the D1 Now Study | Meeting and Delphi methodology | Data analysis, co-authoring |
| Piercy, 2019[71] | United Kingdom | What are the information needs of parents caring for a child with Glutaric aciduria type 1? | Focus group | Research ideas |
| Porcheret, 2013[72] | United Kingdom | Developing a model osteoarthritis consultation: a Delphi consensus exercise | Survey and Delphi consensus technique | Research ideas |
| Priest, 2016[73] | United States | Finding the Patient's Voice Using Big Data: Analysis of Users' Health-Related Concerns in the ChaCha Question-and-Answer Service (2009-2012) | Social media analysis | Research ideas. |
| Puppo, 2020[74] | France | How PrEP users constitute a community in the MSM population through their specific experience and management of stigmatization. The example of the French ANRS-PREVENIR study | Focus group | Research ideas |
| Rankin, 2020[75] | United Kingdom | Identifying Priorities for Physiotherapy Research in the UK: the James Lind Alliance Physiotherapy Priority Setting Partnership | James Lind Alliance method | Research ideas and priorities |
| Rawson, 2018[76] | United Kingdom | Involving citizens in priority setting for public health research: Implementation in infection research | Focus group and meeting | Voting for priorities of research, data analysis |
| Read, 2020[77] | United Kingdom | Facilitating personal development for public involvement in health-care education and research: A co-produced pilot study in one UK higher education institute | Focus groups and  workshops | All stages of the research cycle |
| Robinson, 2020[78] | United Kingdom and Australia | Flipping the paradigm: a qualitative exploration of research translation centres in the United Kingdom and Australia | Semi-structured interviews | Research ideas |
| Russell, 2018[79] | United Kingdom | Selective patient and public involvement: The promise and perils of pharmaceutical intervention for autism | Meeting and survey | Research ideas |
| Sin, 2019[80] | United Kingdom | A Multicomponent eHealth Intervention for Family Carers for People Affected by Psychosis: A Coproduced Design and Build Study | Focus group and workshops | Co-design.  Advisory group |
| Stevenson, 2019[81] | Ireland | Involving individuals with dementia as co-researchers in analysis of findings from a qualitative study | Interview | Data analysis |
| Synnot, 2018[82] | Australia | Research priorities in health communication and participation: international survey of consumers and other stakeholders | James Lind Alliance method | Research ideas,  study scope, co-design, recruitment, interpretation of results and dissemination |
| Tapp, 2017[83] | United States | Patient perspectives on engagement in shared decision-making for asthma care | Meeting and focus group | Co-design research plan, design of instruments, co-authoring and advocacy |
| Taylor, 2015[84] | United Kingdom | Novel participatory methods of involving patients in research: naming and branding a longitudinal cohort study, BRIGHTLIGHT | Workshop | Research ideas |
| Thomas, 2020[85] | United Kingdom | An engaged approach to exploring issues around poverty and mental health: A reflective evaluation of the research process from researchers and community partners involved in the DeStress study | Focus group and workshops | Advisory board, co-author article, project coordinator, organiser conference, presenter in conference |
| Tran, 2019[86] | France | Patients' perspective on how to improve the care of people with chronic conditions in France: a citizen science study within the ComPaRe e-cohort | Survey | Research ideas, data analysis and co-writing the draft |
| Troya, 2019[87] | United Kingdom | Understanding self-harm in older adults: A qualitative study | Interview | Recruitment strategies, data analysis, and dissemination |
| Tsianakas, 2012 [88] | United Kingdom | Using patients’ experiences to identify priorities for quality improvement in breast cancer care: patient narratives, surveys or both? | Survey and interview | Priorities |
| Valaitis, 2019[89] | Canada | Health TAPESTRY: co-designing interprofessional primary care programs for older adults using the persona-scenario method | Workshop | Ideas for health intervention and co-design |
| Vasilica, 2020[90] | United Kingdom | A Co-Designed Social Media Intervention to Satisfy Information Needs and Improve Outcomes of Patients With Chronic Kidney Disease: Longitudinal Study | Social media, survey, interviews and meetings | Co-design |
| Walsh, 2020[91] | Ireland | Taking guidance from parents involved in a longitudinal birth cohort - the ROLO family advisory committee | Social media, survey and meeting | Research ideas |
| Westfall, 2006[92] | United States | Community-based participatory research in practice-based research networks | Survey | Research ideas, review protocols, interpret results, and disseminate findings |
| Wittmeier, 2018[93] | Canada | Identifying Information Needs for Hirschsprung Disease Through Caregiver Involvement via Social Media: A Prioritization Study and Literature Review | Social media, survey | Research ideas, co-design and writing article |
| Woods, 2019[94] | Australia | Co-Design of a Mobile Health App for Heart Failure: Perspectives from the Team | Semi-structured interview | Co-design |
| Woodward, 2016[95] | United Kingdom | An innovative and collaborative partnership between patients with rare disease and industry-supported registries: the Global aHUS Registry | Meeting and survey | Research ideas |
| Young, 2019[96] | United Kingdom | Co-producing Progression Criteria for Feasibility Studies: A Partnership between Patient Contributors, Clinicians and Researchers | Workshop | Co-design, feasibility of an RCT, priorities |
| Zimbudzi, 2019[97] | Australia | The impact of an integrated diabetes and kidney service on patients, primary and specialist health professionals in Australia: A qualitative study | Focus group | Research ideas |

**References**

1. Bayliss K, Starling B, Raza K, Johansson EC, Zabalan C, Moore S, et al. Patient involvement in a qualitative meta-synthesis: lessons learnt. Research involvement and engagement. 2016;2(1):18.

2. Bell T, Vat LE, McGavin C, Keller M, Getchell L, Rychtera A, et al. Co-building a patient-oriented research curriculum in Canada. Research involvement and engagement. 2019;5(1):7.

3. Berwick DM, Loehrer S, Gunther-Murphy C. Breaking the Rules for Better Care. JAMA. 2017 Jun 6;317(21):2161-2. PMID: 28448652. doi: 10.1001/jama.2017.4703.

4. Best P, Badham J, Corepal R, O'Neill RF, Tully MA, Kee F, et al. Network methods to support user involvement in qualitative data analyses: an introduction to Participatory Theme Elicitation. Trials. 2017 Nov 23;18(1):559. PMID: 29169378. doi: 10.1186/s13063-017-2289-5.

5. Bethell J, Puts MTE, Sattar S, Andrew MK, Choate AS, Clarke B, et al. The Canadian Frailty Priority Setting Partnership: Research Priorities for Older Adults Living with Frailty. Canadian geriatrics journal : CGJ. 2019 Mar;22(1):23-33. PMID: 31501680. doi: 10.5770/cgj.22.336.

6. Birnie KA, Dib K, Ouellette C, Dib MA, Nelson K, Pahtayken D, et al. Partnering For Pain: a Priority Setting Partnership to identify patient-oriented research priorities for pediatric chronic pain in Canada. CMAJ Open. 2019 Oct-Dec;7(4):E654-e64. PMID: 31699686. doi: 10.9778/cmajo.20190060.

7. Boddy K, Cowan K, Gibson A, Britten N. Does funded research reflect the priorities of people living with type 1 diabetes? A secondary analysis of research questions. BMJ open. 2017 Sep 27;7(9):e016540. PMID: 28963289. doi: 10.1136/bmjopen-2017-016540.

8. Boney O, Bell M, Bell N, Conquest A, Cumbers M, Drake S, et al. Identifying research priorities in anaesthesia and perioperative care: final report of the joint National Institute of Academic Anaesthesia/James Lind Alliance Research Priority Setting Partnership. BMJ open. 2015 Dec 16;5(12):e010006. PMID: 26674506. doi: 10.1136/bmjopen-2015-010006.

9. Brainard JS, Al Assaf E, Omasete J, Leach S, Hammer CC, Hunter PR. Forced migrants involved in setting the agenda and designing research to reduce impacts of complex emergencies: combining Swarm with patient and public involvement. Res Involv Engagem. 2017;3:23. PMID: 29142759. doi: 10.1186/s40900-017-0073-z.

10. Brett L, Nguyen AD, Siette J, Dove-Pizarro J, Hourihan F, Georgiou A. The co-design of timely and meaningful information needed to enhance social participation in community aged care services: Think tank proceedings. Australasian journal on ageing. 2020 Mar;39(1):e162-e7. PMID: 31411384. doi: 10.1111/ajag.12706.

11. Brooks H, Irmansyah I, Susanti H, Utomo B, Prawira B, Iskandar L, et al. Evaluating the acceptability of a co-produced and co-delivered mental health public engagement festival: Mental Health Matters, Jakarta, Indonesia. Res Involv Engagem. 2019;5:25. PMID: 31516732. doi: 10.1186/s40900-019-0161-3.

12. Brown K, Dyas J, Chahal P, Khalil Y, Riaz P, Cummings-Jones J. Discovering the research priorities of people with diabetes in a multicultural community: a focus group study. Br J Gen Pract. 2006;56(524):206-13.

13. Buffel T. Social research and co-production with older people: Developing age-friendly communities. J Aging Stud. 2018 Mar;44:52-60. PMID: 29502790. doi: 10.1016/j.jaging.2018.01.012.

14. Carr ECJ, Patel JN, Ortiz MM, Miller JL, Teare SR, Barber CEH, et al. Co-design of a patient experience survey for arthritis central intake: an example of meaningful patient engagement in healthcare design. BMC health services research. 2019 Jun 4;19(1):355. PMID: 31164176. doi: 10.1186/s12913-019-4196-9.

15. Casanova T, Black C, Rafiq S, Hugill-Jones J, Read JCA, Vancleef K. The impact of active research involvement of young children in the design of a new stereotest. Res Involv Engagem. 2020;6:29. PMID: 32518689. doi: 10.1186/s40900-020-00194-6.

16. Cheraghi-Sohi S, Hole AR, Mead N, McDonald R, Whalley D, Bower P, et al. What patients want from primary care consultations: a discrete choice experiment to identify patients' priorities. Ann Fam Med. 2008 Mar-Apr;6(2):107-15. PMID: 18332402. doi: 10.1370/afm.816.

17. Costello W, Dorris E. Laying the groundwork: Building relationships for public and patient involvement in pre-clinical paediatric research. Health Expect. 2020 Feb;23(1):96-105. PMID: 31625656. doi: 10.1111/hex.12972.

18. Coultas C, Kieslich K, Littlejohns P. Patient and public involvement in priority-setting decisions in England's Transforming NHS: An interview study with Clinical Commissioning Groups in South London sustainability transformation partnerships. Health Expect. 2019 Dec;22(6):1223-30. PMID: 31410967. doi: 10.1111/hex.12948.

19. Coupe N, Mathieson A. Patient and public involvement in doctoral research: Impact, resources and recommendations. Health Expect. 2020 Feb;23(1):125-36. PMID: 31613049. doi: 10.1111/hex.12976.

20. Dahm MR, Brown A, Martin D, Williams M, Osborne B, Basseal J, et al. Interaction and innovation: practical strategies for inclusive consumer-driven research in health services. BMJ open. 2019 Dec 16;9(12):e031555. PMID: 31848163. doi: 10.1136/bmjopen-2019-031555.

21. Dawson S, Ruddock A, Parmar V, Morris R, Cheraghi-Sohi S, Giles S, et al. Patient and public involvement in doctoral research: reflections and experiences of the PPI contributors and researcher. Res Involv Engagem. 2020;6:23. PMID: 32426162. doi: 10.1186/s40900-020-00201-w.

22. Dewa LH, Lawrence-Jones A, Crandell C, Jaques J, Pickles K, Lavelle M, et al. Reflections, impact and recommendations of a co-produced qualitative study with young people who have experience of mental health difficulties. Health Expect. 2020 Jun 9. PMID: 32515538. doi: 10.1111/hex.13088.

23. Dias S, Gama A, Simões D, Mendão L. Implementation Process and Impacts of a Participatory HIV Research Project with Key Populations. Biomed Res Int. 2018;2018:5845218. PMID: 29955605. doi: 10.1155/2018/5845218.

24. Eccles A, Bryce C, Turk A, Atherton H. Patient and public involvement mobile workshops - convenient involvement for the un-usual suspects. Res Involv Engagem. 2018;4:38. PMID: 30386631. doi: 10.1186/s40900-018-0123-1.

25. Evans BA, Porter A, Snooks H, Burholt V. A co-produced method to involve service users in research: the SUCCESS model. BMC medical research methodology. 2019;19(1):34.

26. Finer S, Robb P, Cowan K, Daly A, Shah K, Farmer A. Setting the top 10 research priorities to improve the health of people with Type 2 diabetes: a Diabetes UK–James Lind Alliance Priority Setting Partnership. Diabetic Medicine. 2018;35(7):862-70.

27. Fonseka TM, Pong JT, Kcomt A, Kennedy SH, Parikh SV. Collaborating with individuals with lived experience to adapt CANMAT clinical depression guidelines into a patient treatment guide: The CHOICE-D co-design process. J Eval Clin Pract. 2020 Aug;26(4):1259-69. PMID: 31729117. doi: 10.1111/jep.13308.

28. Frith L, Hepworth L, Lowers V, Joseph F, Davies E, Gabbay M. Role of public involvement in the Royal College of Physicians’ Future Hospitals healthcare improvement programme: an evaluation. BMJ open. 2019;9(9):e027680.

29. Frost J, Gibson A, Harris-Golesworthy F, Harris J, Britten N. Patient involvement in qualitative data analysis in a trial of a patient-centred intervention: Reconciling lay knowledge and scientific method. Health Expect. 2018 Dec;21(6):1111-21. PMID: 30073734. doi: 10.1111/hex.12814.

30. Gadsby R, Snow R, Daly AC, Crowe S, Matyka K, Hall B, et al. Setting research priorities for Type 1 diabetes. Diabetic medicine : a journal of the British Diabetic Association. 2012 Oct;29(10):1321-6. PMID: 22823450. doi: 10.1111/j.1464-5491.2012.03755.x.

31. Garfield S, Jheeta S, Jacklin A, Bischler A, Norton C, Franklin BD. Patient and public involvement in data collection for health services research: a descriptive study. Res Involv Engagem. 2015;1:8. PMID: 29062497. doi: 10.1186/s40900-015-0006-7.

32. Garfield S, Jheeta S, Husson F, Jacklin A, Bischler A, Norton C, et al. Lay involvement in the analysis of qualitative data in health services research: a descriptive study. Res Involv Engagem. 2016;2:29. PMID: 29507764. doi: 10.1186/s40900-016-0041-z.

33. Ghisoni M, Wilson CA, Morgan K, Edwards B, Simon N, Langley E, et al. Priority setting in research: user led mental health research. Res Involv Engagem. 2017;3:4. PMID: 29062529. doi: 10.1186/s40900-016-0054-7.

34. Gillard S, Simons L, Turner K, Lucock M, Edwards C. Patient and public involvement in the coproduction of knowledge: reflection on the analysis of qualitative data in a mental health study. Qual Health Res. 2012 Aug;22(8):1126-37. PMID: 22673090. doi: 10.1177/1049732312448541.

35. Grant C, Widnall E, Cross L, Simonoff E, Downs J. Informing the development of an E-platform for monitoring wellbeing in schools: involving young people in a co-design process. Res Involv Engagem. 2020;6:51. PMID: 32908677. doi: 10.1186/s40900-020-00219-0.

36. Gregg A, Getz N, Benger J, Anderson A. A Novel Collaborative Approach to Building Better Clinical Trials: New Insights From a Patient Engagement Workshop to Propel Patient-Centricity Forward. Ther Innov Regul Sci. 2019 May 22:2168479019849875. PMID: 31117820. doi: 10.1177/2168479019849875.

37. Grundy A, Keetharuth AD, Barber R, Carlton J, Connell J, Taylor Buck E, et al. Public involvement in health outcomes research: lessons learnt from the development of the recovering quality of life (ReQoL) measures. Health and quality of life outcomes. 2019 Apr 11;17(1):60. PMID: 30975153. doi: 10.1186/s12955-019-1123-z.

38. Hatton AL, Haslam C, Bell S, Langley J, Woolrych R, Cory C, et al. Innovative solutions to enhance safe and green environments for ageing well using co-design through patient and public involvement. Res Involv Engagem. 2020;6:45. PMID: 32760595. doi: 10.1186/s40900-020-00223-4.

39. Higgins PS, Shugrue N, Ruiz K, Robison J. Medicare and Medicaid users speak out about their health care: the real, the ideal, and how to get there. Population health management. 2015;18(2):123-30.

40. Howe A, Mathie E, Munday D, Cowe M, Goodman C, Keenan J, et al. Learning to work together - lessons from a reflective analysis of a research project on public involvement. Res Involv Engagem. 2017;3:1. PMID: 29062526. doi: 10.1186/s40900-016-0051-x.

41. Hyatt A, Morkunas B, Davey D, Thai AA, Trewhella M, Duffy M, et al. Co-design and development of online video resources about immunotherapy with patients and their family. Patient education and counseling. 2020 Sep 16. PMID: 32988686. doi: 10.1016/j.pec.2020.09.014.

42. Irving A, Turner J, Marsh M, Broadway-Parkinson A, Fall D, Coster J, et al. A coproduced patient and public event: An approach to developing and prioritizing ambulance performance measures. Health Expect. 2018 Feb;21(1):230-8. PMID: 28841272. doi: 10.1111/hex.12606.

43. Jennings H, Slade M, Bates P, Munday E, Toney R. Best practice framework for Patient and Public Involvement (PPI) in collaborative data analysis of qualitative mental health research: methodology development and refinement. BMC psychiatry. 2018 Jun 28;18(1):213. PMID: 29954373. doi: 10.1186/s12888-018-1794-8.

44. Jilka S, Murray C, Wieczorek A, Griffiths H, Wykes T, McShane R. Exploring patients' and carers' views about the clinical use of ketamine to inform policy and practical decisions: mixed-methods study. BJPsych Open. 2019 Jul 30;5(5):e62. PMID: 31530293. doi: 10.1192/bjo.2019.52.

45. Jørgensen CR, Eskildsen NB, Johnsen AT. User involvement in a Danish project on the empowerment of cancer patients - experiences and early recommendations for further practice. Res Involv Engagem. 2018;4:26. PMID: 30123531. doi: 10.1186/s40900-018-0105-3.

46. Kalot MA, Al‐Khatib M, Connell NT, Flood V, Brignardello‐Petersen R, James P, et al. An international survey to inform priorities for new guidelines on von Willebrand disease. Haemophilia. 2019.

47. Kelemen M, Surman E, Dikomitis L. Cultural animation in health research: An innovative methodology for patient and public involvement and engagement. Health Expect. 2018 Aug;21(4):805-13. PMID: 29532582. doi: 10.1111/hex.12677.

48. King C, Gillard S. Bringing together coproduction and community participatory research approaches: Using first person reflective narrative to explore coproduction and community involvement in mental health research. Health Expect. 2019 Aug;22(4):701-8. PMID: 31187556. doi: 10.1111/hex.12908.

49. Knowles S, Hays R, Senra H, Bower P, Locock L, Protheroe J, et al. Empowering people to help speak up about safety in primary care: Using codesign to involve patients and professionals in developing new interventions for patients with multimorbidity. Health Expect. 2018 Apr;21(2):539-48. PMID: 29266797. doi: 10.1111/hex.12648.

50. Kuluski K, Ho JW, Cadel L, Shearkhani S, Levy C, Marcinow M, et al. An alternate level of care plan: Co-designing components of an intervention with patients, caregivers and providers to address delayed hospital discharge challenges. Health Expect. 2020 Jun 30. PMID: 32602628. doi: 10.1111/hex.13094.

51. Lee C, Tanna N, Blair M, Yusuf Y, Khalief H, Lakhanpaul M. Getting underneath the skin: A community engagement event for optimal vitamin D status in an 'easily overlooked' group. Health Expect. 2019 Oct 11. PMID: 31605450. doi: 10.1111/hex.12978.

52. Leslie M, Khayatzadeh-Mahani A, MacKean G. Recruitment of caregivers into health services research: lessons from a user-centred design study. Res Involv Engagem. 2019;5:17. PMID: 31139432. doi: 10.1186/s40900-019-0150-6.

53. Little P, Everitt H, Williamson I, Warner G, Moore M, Gould C, et al. Preferences of patients for patient centred approach to consultation in primary care: observational study. BMJ (Clinical research ed). 2001;322(7284):468.

54. Locock L, Robert G, Boaz A, Vougioukalou S, Shuldham C, Fielden J, et al. Using a national archive of patient experience narratives to promote local patient-centered quality improvement: an ethnographic process evaluation of ‘accelerated’experience-based co-design. Journal of health services research & policy. 2014;19(4):200-7.

55. Locock L, Kirkpatrick S, Brading L, Sturmey G, Cornwell J, Churchill N, et al. Involving service users in the qualitative analysis of patient narratives to support healthcare quality improvement. Res Involv Engagem. 2019;5:1. PMID: 30788147. doi: 10.1186/s40900-018-0133-z.

56. Maccarthy J, Guerin S, Wilson AG, Dorris ER. Facilitating public and patient involvement in basic and preclinical health research. PloS one. 2019;14(5):e0216600. PMID: 31086377. doi: 10.1371/journal.pone.0216600.

57. Mackintosh N, Sandall J, Collison C, Carter W, Harris J. Employing the arts for knowledge production and translation: Visualizing new possibilities for women speaking up about safety concerns in maternity. Health Expectations. 2018;21(3):647-58.

58. Madden M, Morris S, Ogden M, Lewis D, Stewart D, McCambridge J. Producing co-production: Reflections on the development of a complex intervention. Health Expect. 2020 Jun;23(3):659-69. PMID: 32233053. doi: 10.1111/hex.13046.

59. Mader LB, Harris T, Klager S, Wilkinson IB, Hiemstra TF. Inverting the patient involvement paradigm: defining patient led research. Res Involv Engagem. 2018;4:21. PMID: 30002875. doi: 10.1186/s40900-018-0104-4.

60. Mann C, Chilcott S, Plumb K, Brooks E, Man MS. Reporting and appraising the context, process and impact of PPI on contributors, researchers and the trial during a randomised controlled trial - the 3D study. Res Involv Engagem. 2018;4:15. PMID: 29785283. doi: 10.1186/s40900-018-0098-y.

61. Mathie E, Smeeton N, Munday D, Rhodes G, Wythe H, Jones J. The role of patient and public involvement leads in facilitating feedback: "invisible work". Res Involv Engagem. 2020;6:40. PMID: 32676199. doi: 10.1186/s40900-020-00209-2.

62. McCarron TL, Noseworthy T, Moffat K, Wilkinson G, Zelinsky S, White D, et al. Understanding the motivations of patients: A co-designed project to understand the factors behind patient engagement. Health Expect. 2019 Aug;22(4):709-20. PMID: 31379094. doi: 10.1111/hex.12942.

63. McCarron TL, Noseworthy T, Moffat K, Wilkinson G, Zelinsky S, White D, et al. A co-designed framework to support and sustain patient and family engagement in health-care decision making. Health Expect. 2020 Aug;23(4):825-36. PMID: 32337836. doi: 10.1111/hex.13054.

64. Mulvale G, Green J, Miatello A, Cassidy AE, Martens T. Finding harmony within dissonance: Engaging patients, family/caregivers and service providers in research to fundamentally restructure relationships through integrative dynamics. Health Expect. 2020 Jun 11. PMID: 32529748. doi: 10.1111/hex.13063.

65. Murta FR, Waxman J, Skilton A, Wickwar S, Bonstein K, Cable R, et al. The first UK national blepharospasm patient and public involvement day; identifying priorities. Orbit. 2019 Aug 30:1-8. PMID: 31466502. doi: 10.1080/01676830.2019.1657469.

66. Neville C, Da Costa D, Mill C, Rochon M, Aviña-Zubieta JA, Pineau CA, et al. The needs of persons with lupus and health care providers: a qualitative study aimed toward the development of the Lupus Interactive Navigator™. Lupus. 2014;23(2):176-82. PMID: 24335012. doi: 10.1177/0961203313517154.

67. Ní Shé É, Cassidy J, Davies C, De Brún A, Donnelly S, Dorris E, et al. Minding the gap: identifying values to enable public and patient involvement at the pre-commencement stage of research projects. Res Involv Engagem. 2020;6:46. PMID: 32765898. doi: 10.1186/s40900-020-00220-7.

68. Nissen ER, Bregnballe V, Mehlsen MY, Muldbjerg A, O'Connor M, Lomborg KE. Patient involvement in the development of a psychosocial cancer rehabilitation intervention: evaluation of a shared working group with patients and researchers. Res Involv Engagem. 2018;4:24. PMID: 30123530. doi: 10.1186/s40900-018-0106-2.

69. O'Donnell D, Ní Shé É, McCarthy M, Thornton S, Doran T, Smith F, et al. Enabling public, patient and practitioner involvement in co-designing frailty pathways in the acute care setting. BMC health services research. 2019 Nov 5;19(1):797. PMID: 31690304. doi: 10.1186/s12913-019-4626-8.

70. O'Hara MC, Hynes L, O'Donnell M, Keighron C, Allen G, Caulfield A, et al. Strength in Numbers: an international consensus conference to develop a novel approach to care delivery for young adults with type 1 diabetes, the D1 Now Study. Res Involv Engagem. 2017;3:25. PMID: 29214056. doi: 10.1186/s40900-017-0076-9.

71. Piercy H, Yeo M, Yap S, Hart AR. What are the information needs of parents caring for a child with Glutaric aciduria type 1? BMC Pediatr. 2019 Oct 13;19(1):349. PMID: 31607269. doi: 10.1186/s12887-019-1742-x.

72. Porcheret M, Grime J, Main C, Dziedzic K. Developing a model osteoarthritis consultation: a Delphi consensus exercise. BMC Musculoskelet Disord. 2013 Jan 16;14:25. PMID: 23320630. doi: 10.1186/1471-2474-14-25.

73. Priest C, Knopf A, Groves D, Carpenter JS, Furrey C, Krishnan A, et al. Finding the Patient's Voice Using Big Data: Analysis of Users' Health-Related Concerns in the ChaCha Question-and-Answer Service (2009-2012). Journal of medical Internet research. 2016;18(3):e44-e. PMID: 26960745. doi: 10.2196/jmir.5033.

74. Puppo C, Spire B, Morel S, Génin M, Béniguel L, Costagliola D, et al. How PrEP users constitute a community in the MSM population through their specific experience and management of stigmatization. The example of the French ANRS-PREVENIR study. AIDS Care. 2020 May;32(sup2):32-9. PMID: 32174136. doi: 10.1080/09540121.2020.1742863.

75. Rankin G, Summers R, Cowan K, Barker K, Button K, Carroll SP, et al. Identifying Priorities for Physiotherapy Research in the UK: the James Lind Alliance Physiotherapy Priority Setting Partnership. Physiotherapy. 2020 Jun;107:161-8. PMID: 32026816. doi: 10.1016/j.physio.2019.07.006.

76. Rawson TM, Castro-Sanchez E, Charani E, Husson F, Moore LSP, Holmes AH, et al. Involving citizens in priority setting for public health research: Implementation in infection research. Health Expect. 2018 Feb;21(1):222-9. PMID: 28732138. doi: 10.1111/hex.12604.

77. Read S, Aries AM, Ashby SM, Bambrick V, Blackburn SJ, Clifford H, et al. Facilitating personal development for public involvement in health-care education and research: A co-produced pilot study in one UK higher education institute. Health Expect. 2020 Jul 24. PMID: 32707602. doi: 10.1111/hex.13097.

78. Robinson T, Skouteris H, Burns P, Melder A, Bailey C, Croft C, et al. Flipping the paradigm: a qualitative exploration of research translation centres in the United Kingdom and Australia. Health Res Policy Syst. 2020 Sep 29;18(1):111. PMID: 32993658. doi: 10.1186/s12961-020-00622-9.

79. Russell G, Starr S, Elphick C, Rodogno R, Singh I. Selective patient and public involvement: The promise and perils of pharmaceutical intervention for autism. Health Expect. 2018 Apr;21(2):466-73. PMID: 29090494. doi: 10.1111/hex.12637.

80. Sin J, Henderson C, Woodham LA, Sesé Hernández A, Gillard S. A Multicomponent eHealth Intervention for Family Carers for People Affected by Psychosis: A Coproduced Design and Build Study. Journal of medical Internet research. 2019 Aug 6;21(8):e14374. PMID: 31389333. doi: 10.2196/14374.

81. Stevenson M, Taylor BJ. Involving individuals with dementia as co-researchers in analysis of findings from a qualitative study. Dementia (London). 2019 Feb;18(2):701-12. PMID: 28133983. doi: 10.1177/1471301217690904.

82. Synnot A, Bragge P, Lowe D, Nunn JS, O'Sullivan M, Horvat L, et al. Research priorities in health communication and participation: international survey of consumers and other stakeholders. BMJ open. 2018 May 8;8(5):e019481. PMID: 29739780. doi: 10.1136/bmjopen-2017-019481.

83. Tapp H, Derkowski D, Calvert M, Welch M, Spencer S. Patient perspectives on engagement in shared decision-making for asthma care. Family practice. 2017;34(3):353-7. PMID: 28034918. doi: 10.1093/fampra/cmw122.

84. Taylor RM, Mohain J, Gibson F, Solanki A, Whelan J, Fern LA. Novel participatory methods of involving patients in research: naming and branding a longitudinal cohort study, BRIGHTLIGHT. BMC Med Res Methodol. 2015 Mar 14;15:20. PMID: 25888163. doi: 10.1186/s12874-015-0014-1.

85. Thomas F, Hansford L, Wyatt K, Byng R, Coombes K, Finch J, et al. An engaged approach to exploring issues around poverty and mental health: A reflective evaluation of the research process from researchers and community partners involved in the DeStress study. Health Expect. 2020 May 24. PMID: 32449304. doi: 10.1111/hex.13065.

86. Tran VT, Riveros C, Pean C, Czarnobroda A, Ravaud P. Patients' perspective on how to improve the care of people with chronic conditions in France: a citizen science study within the ComPaRe e-cohort. BMJ Qual Saf. 2019 Apr 23. PMID: 31015376. doi: 10.1136/bmjqs-2018-008593.

87. Troya MI, Dikomitis L, Babatunde OO, Bartlam B, Chew-Graham CA. Understanding self-harm in older adults: A qualitative study. EClinicalMedicine. 2019.

88. Tsianakas V, Maben J, Wiseman T, Robert G, Richardson A, Madden P, et al. Using patients’ experiences to identify priorities for quality improvement in breast cancer care: patient narratives, surveys or both? BMC health services research. 2012;12(1):271.

89. Valaitis R, Longaphy J, Ploeg J, Agarwal G, Oliver D, Nair K, et al. Health TAPESTRY: co-designing interprofessional primary care programs for older adults using the persona-scenario method. BMC family practice. 2019 Sep 4;20(1):122. PMID: 31484493. doi: 10.1186/s12875-019-1013-9.

90. Vasilica CM, Brettle A, Ormandy P. A Co-Designed Social Media Intervention to Satisfy Information Needs and Improve Outcomes of Patients With Chronic Kidney Disease: Longitudinal Study. JMIR Form Res. 2020 Jan 27;4(1):e13207. PMID: 32012040. doi: 10.2196/13207.

91. Walsh NM, O'Brien EC, Geraghty AA, Byrne DF, Whelan A, Reilly S, et al. Taking guidance from parents involved in a longitudinal birth cohort - the ROLO family advisory committee. Res Involv Engagem. 2020;6:16. PMID: 32368350. doi: 10.1186/s40900-020-00200-x.

92. Westfall JM, VanVorst RF, Main DS, Herbert C. Community-based participatory research in practice-based research networks. Ann Fam Med. 2006 Jan-Feb;4(1):8-14. PMID: 16449391. doi: 10.1370/afm.511.

93. Wittmeier KD, Hobbs-Murison K, Holland C, Crawford E, Loewen H, Morris M, et al. Identifying Information Needs for Hirschsprung Disease Through Caregiver Involvement via Social Media: A Prioritization Study and Literature Review. Journal of medical Internet research. 2018 Dec 21;20(12):e297. PMID: 30578208. doi: 10.2196/jmir.9701.

94. Woods L, Roehrer E, Duff J, Walker K, Cummings E. Co-Design of a Mobile Health App for Heart Failure: Perspectives from the Team. Studies in health technology and informatics. 2019 Aug 8;266:183-8. PMID: 31397321. doi: 10.3233/shti190792.

95. Woodward L, Johnson S, Walle JV, Beck J, Gasteyger C, Licht C, et al. An innovative and collaborative partnership between patients with rare disease and industry-supported registries: the Global aHUS Registry. Orphanet J Rare Dis. 2016 Nov 21;11(1):154. PMID: 27871301. doi: 10.1186/s13023-016-0537-5.

96. Young HML, Goodliffe S, Madhani M, Phelps K, Regen E, Locke A, et al. Co-producing Progression Criteria for Feasibility Studies: A Partnership between Patient Contributors, Clinicians and Researchers. International journal of environmental research and public health. 2019 Oct 6;16(19). PMID: 31590424. doi: 10.3390/ijerph16193756.

97. Zimbudzi E, Lo C, Robinson T, Ranasinha S, Teede HJ, Usherwood T, et al. The impact of an integrated diabetes and kidney service on patients, primary and specialist health professionals in Australia: A qualitative study. PloS one. 2019;14(7):e0219685. PMID: 31306453. doi: 10.1371/journal.pone.0219685.
