# Supplementary material for: Methods to Generate Innovative Research Ideas and Improve Patient and Public Involvement in Modern Epidemiological Research: Review, Patient Viewpoint, and Guidelines for Implementation of a Digital Cohort Study
Source: J Med Internet Res. 2021 Dec 23;23(12):e25743. doi: 10.2196/25743 (PMC8738987; doi:10.2196/25743)
Supplement: Multimedia Appendix 2 [file jmir_v23i12e25743_app2.docx]

## Multimedia Appendix 2. A focus group guide

An organized outline of the focus group interview used in a E-cohort study implementation phase is presented below. The questions are in a logical flow and time sequence, but they can be adapted to better fit the purposes of the new research to develop.

**FOCUS GROUP INTERVIEW GUIDE**

**Welcome**

Thanks for agreeing to be part of the E-cohort study focus group. We appreciate your willingness to participate.

**Introduction**

Moderator and assistant moderator

**Purpose**

We have been asked by the E-cohort study project to conduct these focus groups.

We would like to understand patients’ perceptions regarding their participation in the E-cohort study.

We need your input and want you to share your honest and open thoughts with us.

**Presentation of the flyer and the website**

Does seeing this flyer make you want to participate? Why?

Does seeing this website make you want to participate? Why?

**Presentation of the objectives**

Are the objectives clear enough? Why?

Does it make you want to participate? Why?

**Data collection in E-cohort study**

Would you be willing to share your data?

If yes, how?

If no, why and which type of data would you refuse?

**Terms of participation**

If you agree to participate, how many times per month would you agree to be contacted?

What frequency of contact?

Could we resend invitations in the event of non-response?

Do you prefer to be contacted during the week, the weekend, the evening or during the day?

**Expectations for participation**

Would you like to receive feedback?

Would you like to receive study results as a preferred receiver?

Would you like to participate in events organized by the E-cohort study?

Would you like to participate in E-cohort study's online communities?

**Patient involvement**

Would you like to be consulted/involved in the generation of ideas for future diabetes research?

Would you like to become an E-cohort study Ambassador? (this means for example to represent the project in patient associations or shoot a short video)

Would you like to become a patient-researcher developing your own projects supported by the E-cohort study research team?
