# Supplementary material for: Methods to Generate Innovative Research Ideas and Improve Patient and Public Involvement in Modern Epidemiological Research: Review, Patient Viewpoint, and Guidelines for Implementation of a Digital Cohort Study
Source: J Med Internet Res. 2021 Dec 23;23(12):e25743. doi: 10.2196/25743 (PMC8738987; doi:10.2196/25743)
Supplement: Multimedia Appendix 3 [file jmir_v23i12e25743_app3.docx]

## Multimedia Appendix 3. Grid for assessment of a smartphone application

Dear participant, before I start, I will quickly remind you the purpose of this activity. We would like to collect information about the user experience of a new research app. This information will help us improve this tool for the community of people living with the disease.

You will receive an iPad with a new application, called XX. You will also receive login credentials to this application. You will then have to test the application against the ease or difficulty of the following tasks:

**1. Log in**

**2. Create your profile**

**3. Respond to a questionnaire**

**4. Record your voice in the app**

**5. Take a selfie in the app**

While you are using the app, we will measure the time and note some observations of the difficulties detected.

Then, I am going to ask you an open question, which you can answer very freely, developing your answers as much as you want. There are no right or wrong answers; it is your own point of view that interests me.

Please note that I have no connection with your healthcare team and that the information you can tell me during this interview will never be communicated to them. Your response will be recorded, with your consent, for the purposes of analysis. The recording will be used anonymously and only for the purposes of analysis and will be kept confidential and may not be released under any circumstances.

(See grid in next page)

**Observation grid while using the iPad**

|  | **Result**  1. Successful;  2. Not successful | ***Difficulty***  *1. No difficulty 2. Was successful, but with some difficulties / hesitation 3. Has succeeded with great difficulty  4. Did not succeed alone / Asked for help* | **Time** (sec) | **Observations** |
| --- | --- | --- | --- | --- |
| **Login** |  |  |  |  |
| **Profile** |  |  |  |  |
| **Questionnaire** |  |  |  |  |
| **Voice** |  |  |  |  |
| **Selfie** |  |  |  |  |

Can you briefly tell us about your experience with the app? (Positive why? Less positive, why?)

**……………………………………………………………………………………………………………………………………………………………………………………………………………………………………………………………………………………………………………………………………………………………………………………………………………………………………………………………………………………**
